# Chemokine Function in Periodontal Disease and Oral Cavity Cancer

**DOI:** 10.3389/fimmu.2015.00214

**Published:** 2015-05-05

**Authors:** Sinem Esra Sahingur, W. Andrew Yeudall

**Affiliations:** ^1^Department of Periodontics, Virginia Commonwealth University, Richmond, VA, USA; ^2^Department of Microbiology and Immunology, Virginia Commonwealth University, Richmond, VA, USA; ^3^Department of Oral and Craniofacial Molecular Biology, Virginia Commonwealth University, Richmond, VA, USA; ^4^Department of Biochemistry and Molecular Biology, Virginia Commonwealth University, Richmond, VA, USA; ^5^Massey Cancer Center, Virginia Commonwealth University, Richmond, VA, USA

**Keywords:** chemokine, periodontitis, inflammation, oral cancer, host–pathogen interactions, Toll-like receptor

## Abstract

The chemotactic cytokines, or chemokines, comprise a superfamily of polypeptides with a wide range of activities that include recruitment of immune cells to sites of infection and inflammation, as well as stimulation of cell proliferation. As such, they function as antimicrobial molecules and play a central role in host defenses against pathogen challenge. However, their ability to recruit leukocytes and potentiate or prolong the inflammatory response may have profound implications for the progression of oral diseases such as chronic periodontitis, where tissue destruction may be widespread. Moreover, it is increasingly recognized that chronic inflammation is a key component of tumor progression. Interaction between cancer cells and their microenvironment is mediated in large part by secreted factors such as chemokines, and serves to enhance the malignant phenotype in oral and other cancers. In this article, we will outline the biological and biochemical mechanisms of chemokine action in host–microbiome interactions in periodontal disease and in oral cancer, and how these may overlap and contribute to pathogenesis.

## Introduction

The human body is constantly under assault from a plethora of environmental factors that includes chemical, physical, and microbiological agents. In turn, cellular mechanisms have developed to combat these noxious stimuli, and cells of the innate immune system are central players. Sophisticated molecular regulatory pathways exist to coordinate the host response to bacterial infection and other microbiological challenges. Innate immunity also plays a fundamental role in the pathogenesis of malignant disease, acting as a surveillance mechanism to prevent tumor establishment. On the other hand, the cellular immune response is now recognized as a key promoter of tumor progression and metastasis through potentiation of chronic inflammation at tumor sites. Today, it is widely accepted that deregulated inflammation within the cellular microenvironment is one of the key elements driving tumorigenesis (1–4). Therefore, how immune and inflammatory processes are regulated and how they may result in different outcomes to the host are intriguing questions that are beginning to be understood in some detail.

The oral cavity is home to a diverse microbial community of more than 700 microbial species including commensal and opportunistic bacteria, viruses and fungi living in a symbiotic relationship with each other and the host immune system (5–7). The host–microbiome interactions at the oral mucosal surface are critical to maintain periodontal tissue homeostasis, and the balance between microbial cell turnover and host pro-inflammatory and anti-inflammatory responses eventually determines the clinical outcome. Deregulated host immune responses resulting from environmental and systemic exposures (e.g., smoking, obesity, stress, aging, diabetes), host genetic, and epigenetic defects and/or dysbiotic oral microflora subverting the host defense mechanisms lead to chronic periodontitis (8–13). Thus, today it is believed that although microbial insult initiates the periodontal disease, deregulated immune response mechanisms determine the progression of the lesion and the extent of tissue destruction.

Periodontal disease affects 47% of the population (14). It is defined as inflammation of the periodontium involving the supporting tissues of the teeth and it is characterized by loss of epithelial attachment, connective tissue, and alveolar bone. Chronic periodontitis also serves as a constant reservoir of inflammatory mediators and microbial products that can act upon host tissues. Thus, besides its destructive local effects, persistent forms of the disease are also associated with several systemic conditions, including cardiovascular diseases, adverse pregnancy outcomes, rheumatoid arthritis, diabetes, and pulmonary diseases (15–21).

Emerging evidence also suggests a link between periodontitis and oral cancer, the rationale being that chronic inflammation is a major factor in both diseases (22, 23). The ability to re-route immune cells to a site of infection within the body relies in large part on the action of chemotactic cytokines, or chemokines. These are small polypeptides that are secreted into the microenvironment, and which serve to recruit leukocytes and other immunological mediators to their point of action, such as periodontal inflammatory foci. However, as well as these functions, some chemokines and their receptors have been implicated in cancer development and progression by promoting cell proliferation, motility, angiogenesis, and metastatic spread (3).

In this review article, we will outline the pathogenesis of periodontal disease and oral cancer and the plausible biological mechanisms that may link these, focusing on chemokine ligand and receptor function, and how this might promote tumorigenesis through modulation of the microenvironment.

## Periodontal Disease Pathogenesis

The development and progression of periodontitis is a complex process initiated by a dysbiotic polymicrobial insult and involves multiple host cells of myeloid and non-myeloid origin including oral keratinocytes, neutrophil polymorphs (PMNs), macrophages, monocytes, dendritic cells, osteoblasts, and osteoclasts. These cells possess cytosolic, membrane-associated, and secreted pattern recognition receptors (PRRs) including toll-like receptors (TLRs), NOD-like receptors (NLRs), RIG-I-like receptors (RLR), and C-type lectin receptors, which can engage with periodontal microbial associated molecular patterns (MAMPs) [e.g., lipopolysaccharide (LPS), lipoproteins, fimbriae, BspA, nucleic acids] and damage/danger associated molecular patterns (DAMPs) (e.g., nucleic acids, fibrinogen, heat-shock proteins). This interaction releases inflammatory mediators that aid in the development of an efficient innate immune response to eliminate the pathogen and coordinate development of an adaptive immune response. Although activation of the immune system is crucial to combat microbial insult, deregulated, persistent immune responses due to factors that are still not completely understood result in chronic inflammation, eventually leading to periodontal tissue destruction (24).

In its most general classification, periodontal disease can be categorized as either *gingivitis* or *periodontitis*. Gingivitis defines the inflammation of gingival tissues without alveolar bone loss, whereas periodontitis is accompanied by destruction of alveolar bone. Periodontal disease progression follows four histological stages: *initial*, *early*, *established*, and *advanced* (25). These definitions are based on the distinctive histological features of the pathological site with regard to the cells and the extent of tissue destruction involved. However, it is important to note that disease progression is a dynamic and highly interactive process where there is overlap between each stage, with common cells and inflammatory mediators.

The *initial* sequela to the polymicrobial insult is an acute inflammatory response with increased vascular dilation and blood flow. There is activation of complement and kinin systems and arachidonic acid pathways. There is also increased PMN migration toward the lesion due to chemotactic stimuli originating both from microbial cells as well as host-derived inflammatory mediators. These include IL-8 (CXCL8), complement components C5a and C3, and leukotrines. Some collagen loss is also apparent. The character and intensity of the inflammatory response determine whether the *initial* lesion resolves rapidly, with restoration of the tissue to a normal state, or whether it evolves into a chronic inflammatory lesion.

*Early* lesion is characterized by accentuation of the features of the *initial* lesion with increased vascularization, accumulation of more PMNs and lymphocytes (mainly T-cells), as well as continuous activation of complement and arachidonic acid pathways. Macrophages, plasma cells, and mast cells start to appear at the site of acute inflammation. These cells produce pro-inflammatory mediators including IL-1β, IL-6, IL-8, IL-17, and TNF-α, which may exacerbate the inflammatory response and promote progression to more advanced stages of disease. There is further loss of the collagen fiber network around the inflammatory infiltrate due to activation of the local immune system.

As the disease progresses into the *established* phase, there is a transition from an acute to a chronic state. The manifestations of *early* and *initial* changes still persist, along with the appearance of B-lymphocytes and continuingly increased numbers of PMNs, macrophages, monocytes, and T-cells. There is also the presence of extravascular immunoglobulins in the connective tissue and in the junctional epithelium. In the *established* lesion, which is clinically diagnosed as *chronic gingivitis*, there is substantial loss of gingival extracellular matrix due to increased collagenase activity and activation of the local immune system, but without bone loss.

The *advanced lesion* constitutes the final stage of the transition to periodontitis. At this stage, there is progression of inflammation to involve the alveolar bone. Production of inflammatory mediators such as cytokines, chemokines, arachidonic acid metabolites (prostaglandins), and complement proteins by activated PMNs, macrophages, monocytes, lymphocytes, fibroblasts, and other host cells can cause oxidative damage by promoting the release of tissue-derived enzymes such as matrix metalloproteinases (MMPs). Furthermore, cytokines can act on stromal and non-stromal cells causing increased expression of receptor activator of nuclear factor kappa-B ligand (RANKL) while decreasing osteoprotegerin (OPG) production. If the inflammation is not resolved, destruction of extracellular matrix and irreversible alveolar bone loss occur.

In summary, a polymicrobial insult initiates the periodontal disease process while the progression and clinical presentation of the disease involve complex interactions between host cells and the oral microbiome, triggering persistent inflammation, which eventually destroys the local tissues and further impacts distant sites due to the continuous access of inflammatory mediators and microbial components into the systemic circulation. The biological and biochemical pathways that may link chronic periodontitis with various systemic diseases are reviewed elsewhere (15–21).

## Pathogenesis of Oral Squamous Cell Carcinoma

Squamous cell carcinoma is the most frequently occurring malignancy of the oral cavity and adjacent sites, representing over 90% of all cancers. Worldwide, 200,000 new cases of oral cavity and lip cancer are diagnosed annually, with around 98,000 deaths (http://globocan.iarc.fr/Pages/fact_sheets_population.aspx). The predominant etiological factors for oral cavity cancer are alcohol and tobacco use, with carcinogens impacting on the oral mucosa to create a field that is predisposed to undergo malignant transformation, so-called “field cancerization.” Although, with early detection, “cure” rates may be as high as 50%, most lesions are diagnosed at a late stage and have a much poorer prognosis due to locoregional or distant spread, with 5-year survival as low as 16%. Disappointingly, clinical outcomes have not improved significantly in decades, in spite of advances in surgical, chemotherapeutic, and radiotherapeutic management, as well as the advent of targeted therapies.

The molecular pathogenesis of oral cavity cancer is, in many cases, the result of dysregulation of common signaling pathways that actively drive oncogenesis, on a background of tumor suppressor inactivation. The basis for this may be a combination of somatic mutations, as described recently (26), together with epigenetic and transcriptomic alterations. As just one example, hyperactivation of epidermal growth factor receptor (EGFR) signaling through receptor overexpression, or mutation of either the receptor or downstream signaling components, can lead to enhanced cell proliferation and motility, thereby contributing to tumor growth and metastasis. Inactivation of the *P53* and *CDKN2A* tumor suppressors also occurs with high frequency in oral cancer. In addition to molecular dysregulation as a result of chemical carcinogens in tobacco and alcohol, infectious agents play a role in development and progression of oral cancer. Although considerably less frequent than in the oropharynx, human papillomaviral-related carcinogenesis contributes to a small proportion of oral cavity cancers [around 10%: (26)]. What is less clear, however, is a mechanism that might explain the long-standing notion that poor oral hygiene is related to OSCC development. As in other cancers, though, there is no doubt that chronic inflammatory conditions underpin oral carcinogenesis, and perturbations of cytokine- and chemokine-dependent immunoregulatory pathways are evident in oral cancer. Of course, the oral cavity is prone to a number of bacterial infectious diseases, such as periodontitis, and it is possible that oral bacteria may serve to initiate or promote tumor development, analogous to the association of gastric cancer with *Helicobacter pylori* infection. In fact, a number of periodontal bacteria including *Prevotella intermedia*, *Porphyromonas gingivalis*, *Fusobacterium nucleatum* have been associated with OSCC (27, 28) The plausible biological mechanisms, including microbial and inflammatory, that may link periodontitis and oral cancer are discussed below.

## Chemokines

**Figure 1:**
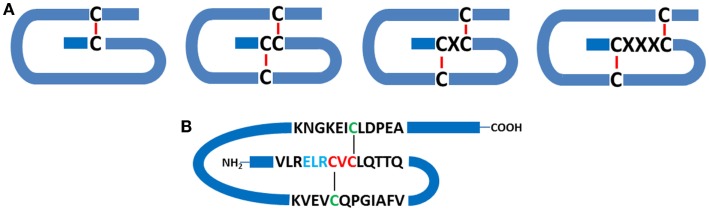
**Chemokine structure**. **(A)** Schematic indicating the relationships between conserved cysteine residues (C), together with intrachain disulfide bridges. C-, CC-, CXC-, and CX_3_C- classes of chemokines are depicted. **(B)** Schematic representation of human CXCL5, a pro-inflammatory and pro-angiogenic ELR^+^ chemokine, which contains the ELR motif N-terminal to the CXC consensus sequence.

Members of the chemokine superfamily are secreted polypeptides, which range in molecular mass from around 5 to 20 kDa. Historically, these were named based on their function (e.g., macrophage inflammatory protein: MIP-1α, β), but a generic nomenclature is now in use. This categorizes chemokines into four subfamilies based on the relative position of conserved cysteine residues within the polypeptide. Thus, C, CC, CXC, and CX_3_C chemokines are recognized (Figure 1).

Chemokine ligands bind to cell surface receptors and the interactions may be unique (single ligand and single receptor) or promiscuous (single ligand/multiple receptors, or multiple ligands/single receptor). For example, IL-8 (CXCL8) binds to both high- and low-affinity receptors (CXCR1 and CXCR2, respectively), whereas CXCL5 only binds to CXCR2. Conversely, the CXCR2 receptor binds multiple CXC-chemokine ligands, whereas CXCR1 binds IL-8 exclusively. Intracellular signaling pathways activated by chemokine ligand-receptor binding include those mediated by extracellular signal regulated kinases (ERKs), phosphoinositide 3-kinase (PI3K), AKT, small GTPases (Rac, Rho, cdc42), and NFκB family members. Thus, there is good potential for molecular crosstalk with growth factor signaling pathways.

In general, the CXC chemokines primarily activate neutrophils, whereas CC chemokines are mainly chemotactic for monocytes/macrophages and lymphocytes, and play a role in the class switching of macrophages from M1 to M2 during the transition from acute to chronic inflammation. CC chemokines are also crucial in the development of adaptive immunity by propagating lymphocyte recruitment and antigen presentation, and they play a critical role in bone metabolism by providing signals for the trafficking of osteoblast and osteoclast precursors.

Several inflammatory mediators including cytokines are abundantly expressed in the course of periodontitis (29). With regards to cancer risk, chemotactic cytokines or chemokines are of particular interest to us as they are involved in almost every stage of periodontal pathogenesis and can also be expressed in healthy sites. In addition to their role in immune cell trafficking to the site of infection, they also regulate angiogenesis, cell proliferation, apoptosis, and tumor cell homing. The next section will give a brief overview of chemokines involved in chronic periodontitis. Later, we will discuss how they may modulate the oral microenvironment to promote cancer progression.

## Chemokines in Periodontal Disease

As noted above, the host immune response to infection is largely regulated by cytokine signals that drive initiation and progression of the inflammatory process. Their involvement with periodontal disease and their possible systemic effects have been well-reviewed elsewhere (24, 30). Here, we will focus specifically on chemokines that are expressed in periodontal tissues and their potential modulatory role in the tumor microenvironment.

Chemokine expression can be triggered by MAMPs, DAMPs, inflammatory mediators, host factors, and mechanical stress. In the oral tissues, chemokines can be synthesized by a variety of cell types including fibroblasts, osteoblasts, endothelial, epithelial and mast cells, PMNs, lymphocytes, and monocytes/macrophages. Thus, they play a major role in immune cell trafficking to sites of periodontal infection (29).

IL-8 (CXCL8) is one of the most abundantly expressed chemokines in the oral cavity and can be detected in both healthy and periodontally diseased tissues (31–36). IL-8 is primarily produced by gingival keratinocytes, fibroblasts, endothelial cells, and macrophages in response to periodontal bacteria and bacterial components. It functions to direct cell trafficking, mainly PMNs, to the site of infection. Inflammatory cell recruitment eventually results in more cytokine production, thus contributing directly to progression of the periodontal lesion. Although clinical studies consistently show that increased IL-8 levels are associated with periodontally diseased sites (31–36), *in vitro* studies with individual periodontal pathogens show variations in IL-8 production depending on the bacteria and the cell type being studied. For example, *P. gingivalis*, a key-stone periodontal pathogen, shows a distinct ability to manipulate local immune responses for its survival, either by increasing or suppressing IL-8 production (37–42). These unique features, possessing both pro- and anti-inflammatory properties, give *P. gingivalis* the ability to create a dysbiotic environment and escape immune surveillance. However, the possible biological mechanisms through which this bacterium may promote tumor development have yet to be investigated in depth.

Besides being a major chemoattractant for PMNs, IL-8 can also affect bone metabolism through its direct actions on osteoclast differentiation and activity by signaling through the high-affinity CXCR1 receptor (43). Additionally, the results of a meta-analysis investigating the risk of oral cancer in patients with polymorphisms of the IL-8-251A >T locus revealed that Caucasian populations harboring the AA genotype had a higher risk of developing malignancy (44), whereas a separate meta-analysis also indicated increased risk for individuals with either the AT or AA genotype (45). The functional significance of this finding is yet to be determined. Thus, many periodontal bacteria and bacterial components can trigger IL-8 production in different cells of the periodontal tissues and IL-8 is considered as one of the major chemokines associated with periodontal disease. Therefore, future investigations are warranted to elucidate the biological mechanisms triggered by IL-8 that modulate the oral environment and increase susceptibility for tumor development and/or progression.

Besides IL-8, another CXC-chemokine, CXCL12 is reported to provide trafficking signals for osteoblast and osteoclast precursors, and it also enhances the activity of MMP9 in osteoclasts, promoting bone resorption. CXCL10, which is a chemoattractant for activated Th1 cells, is found in inflamed gingival tissues (29) and is expressed by human gingival fibroblasts (46) in response to interferon-γ, tumor necrosis factor-α, and IL-1β, suggesting this as a mechanism to recruit Th1 cells to inflammatory foci in periodontitis. Notably, these authors also found that the anti-inflammatory cytokine IL-10 represses CXCL10 expression. B-lymphocytes are also plentiful in periodontal lesions, and it is not surprising that CXCL13, which is chemoattractant for B-cells, is highly expressed in diseased tissues, suggesting a role for this chemokine in the local humoral response to periodontal pathogens.

CC-chemokines are also documented to play various roles in the pathogenesis of periodontitis. CCL2 and CCL3 are chemotactic for monocytes and lymphocytes, CCL4 is chemotactic for CD4^+^ T cells, and CCL5 attracts Th1 cells. All of these ligands have been found at higher levels in chronic periodontitis patients compared to healthy controls, and are associated with disease severity (47–49). Increased expression of the CC-chemokines, CCL2, and CCL20 was also correlated with increased numbers of dendritic cells (50). Macrophages are one of the key cell types found in large numbers in periodontitis lesions, and are involved in phagocytosis of pathogens and production of inflammatory mediators. However, increased accumulation of macrophages and deregulated inflammatory responses can disrupt tissue homeostasis, thereby contributing to periodontal disease. Like IL-8, CCL3 and CCL5 also play roles in bone metabolism and induce migration and activation of osteoclasts. Thus, it is likely that they exacerbate periodontal disease severity.

CCL20 is involved in Th17 cell recruitment and may promote periodontal disease. Hosokawa and coworkers (51) demonstrated that IL-22 increased CCL20 production in human gingival fibroblasts through an NF-κB-dependent mechanism when the cells were pre-challenged with IL-1β. Similarly, IL-6-stimulated periodontal ligament cells showed elevated secretion of CCL20 in a STAT3-dependent manner (52).

CCR4 is a high affinity receptor for both CCL17 (53) and CCL22 (54) and is expressed at higher levels in chronic periodontitis, as well as being associated with higher levels of the anti-inflammatory IL-10 in the periodontium (55). CCL17 and CCL22 are also expressed in diseased periodontium, and it may be possible that expression of these ligands serves to limit periodontal disease severity by attracting Th2 and Treg cells. Stimulation of human gingival fibroblasts with a combination of TNF-α and IL-4/IL-13 was found to increase expression of CCL17. While CCL17 expression was inhibited by *Escherichia coli* LPS and Pam3CSK4, which are activators of TLR4 and TLR2 signaling, respectively, in TNF-α/IL-4 stimulated gingival fibroblasts, CpG DNA (which activates TLR9) enhanced CCL17 production induced by TNF-α/IL-4. Thus, it remains unclear whether CCL17 is likely to reduce or exacerbate inflammation in periodontitis.

## Chemokines in Oral Cancer

Although the normal function of the chemokine system is immunomodulation, there is an ever-increasing body of evidence that documents subversion and dysregulation of this intricate network of signaling molecules during the onset and progression of malignant disease. Overall, altered chemokine function in cancer promotes cell survival, enhanced proliferation, neovascularization, motility and metastasis in multiple tumor types, and this is comprehensively reviewed elsewhere. Several studies have implicated a number of chemokines and their receptors in oral squamous cell carcinogenesis [reviewed in Ref. (56)]. Most frequently, this appears to involve chemokines of the CXC subgroup such as CXCL1 (Gro-α), CXCL8 (IL-8), CXCL5, and CXCL12 (SDF-1). IL-8 has long been recognized as an autocrine regulator of OSCC growth (57), and it also contributes to enhanced cell motility (58). Indeed, salivary IL-8 has been proposed to be a discriminative biomarker for oral cancer (59). The closely related CXCL5 protein, normally chemotactic for neutrophils, has also been shown to drive oral cancer cell growth and motility as well as enhance tumor angiogenesis. Moreover, *in vivo* studies demonstrated complete inhibition of the tumorigenic phenotype when CXCL5 expression was suppressed in OSCC cells (60). Studies by Khurram and colleagues (61) reported elevated expression of both CXCR1 and CXCR2 in oral cancer. The former is a high-affinity IL-8 receptor, and the latter is a low-affinity IL-8 receptor that is also able to bind a number of CXC-chemokines including CXCL1, CXCL2, CXCL3, CXCL5, and CXCL6. These authors reported coincidental activation of signaling pathways by IL-8 and CXCL1, suggesting that simultaneous stimulation of cancer cells by multiple chemokines in the tumor microenvironment is likely to occur.

The CXCL12 (SDF-1)/CXCR4 axis also appears to play critical roles in OSCC development and progression. Expression of the CXCR4 receptor is elevated in tongue cancers (62), with metastatic tumor cells expressing higher levels than non-metastatic. This is consistent with the homing model proposed by Muller et al. (63), where tumor cells that overexpress a chemokine receptor migrate preferentially to organs that express the cognate ligand *via* a chemokine gradient. Perhaps of high relevance to this review, bacterial products have been reported to increase the expression of CXCR4 on oral cancer cells (64).

SDF-1/CXCR4-driven invasion of oral cancer is reported to be dependent upon NFkB signaling (65, 66). Further studies have demonstrated release of the CXCR4 ligand, SDF-1, from bone cells adjacent to the tumor. This has been proposed to contribute to bone turnover as a result of CXCR4-mediated upregulation of IL-6, which is then secreted by the tumor cells to stimulate osteoclastogenesis (67), thereby enhancing tumor invasion. Studies by Oue et al. (68) provided further evidence of a role for IL-6 and RANKL in OSCC, mediated at least in part through a CXCL2-dependent mechanism, as the effects were diminished by a CXCL2 neutralizing antibody.

Of course, there is always the potential for repression of malignant properties by chemokines, as some have noted anti-tumor effects. The chemokine CXCL14, also known as BRAK to signify the tissues (*br*east *a*nd *k*idney) from which it was originally isolated (69), was shown to be expressed in normal squamous epithelium but reduced or absent in malignant oral tissues (70), and is also repressed by EGFR signaling (71). Ectopic expression of CXCL14 completely blocked OSCC growth *in vivo* (72), suggesting that loss of expression may contribute significantly to the pathogenesis of oral cancer.

The CC-class of chemokines has also been implicated in oral carcinogenesis. Studies by Ferris and coworkers (73) documented loss of CCR6 and upregulation of CCR7, the receptor for CCL19 and CCL21, on oral cancer cells, and demonstrated that this was related to lymph node metastasis. CCL5–CCR5 signaling is reported to enhance OSCC motility, as well as increasing production of the gelatinase, MMP9 (74). An interesting study from Li and colleagues (75) demonstrated CCL2 production by cancer-associated fibroblasts (CAFs), which stimulated production of reactive oxygen species (ROS) in cocultured oral cancer cells. In turn, ROS promoted further CCL2 production in CAFs, thus generating a mutually beneficial microenvironment in which both tumor cells and fibroblasts could thrive.

Evidence is also present in the literature to support a role for XCR1, a receptor for lymphotactin (XCL1), in epithelial biology. Khurram and colleagues (76) reported expression of this receptor on oral cancer cells as well as on normal keratinocytes and neutrophils. Based on immunohistochemical analysis, low but detectable expression in normal mucosa was primarily restricted to the basal layer. In contrast, XCR1 expression was more marked in oral cancer tissues and could also be found in metastatic deposits. Interestingly, positive staining was observed in lichen planus, a potentially premalignant condition of immunological etiopathogenesis. Stimulation of XCR1 activity led to elevated expression of the gelatinases MMP2 and MMP9, which was more marked in cancer cells compared to normal keratinocytes. Moreover, cancer cells expressed MMP7, which was not observed in normal cells treated with lymphotactin. Potentially, then, XCR1 may mediate matrix remodeling in oral epithelium, and potentiate migration and invasion in malignant disease.

## Potential Biological and Biochemical Mechanisms Linking Periodontal Disease and Oral Cancer

It has been estimated that 15–20% of tumors are driven by chronic infection and inflammation, and that individuals who are prone to chronic inflammatory disorders have an increased risk of cancer development (77). Recent advances suggest that the tumor microenvironment plays an important role in the initiation and progression of malignant disease (78). This can be mediated through biological factors related to genetic and epigenetic make-up of the cancer cells or through interaction of tumor cells with the surrounding milieu, which is composed of cells and soluble molecules of both microbial and host cell origin as well as extracellular matrix.

**Figure 2:**
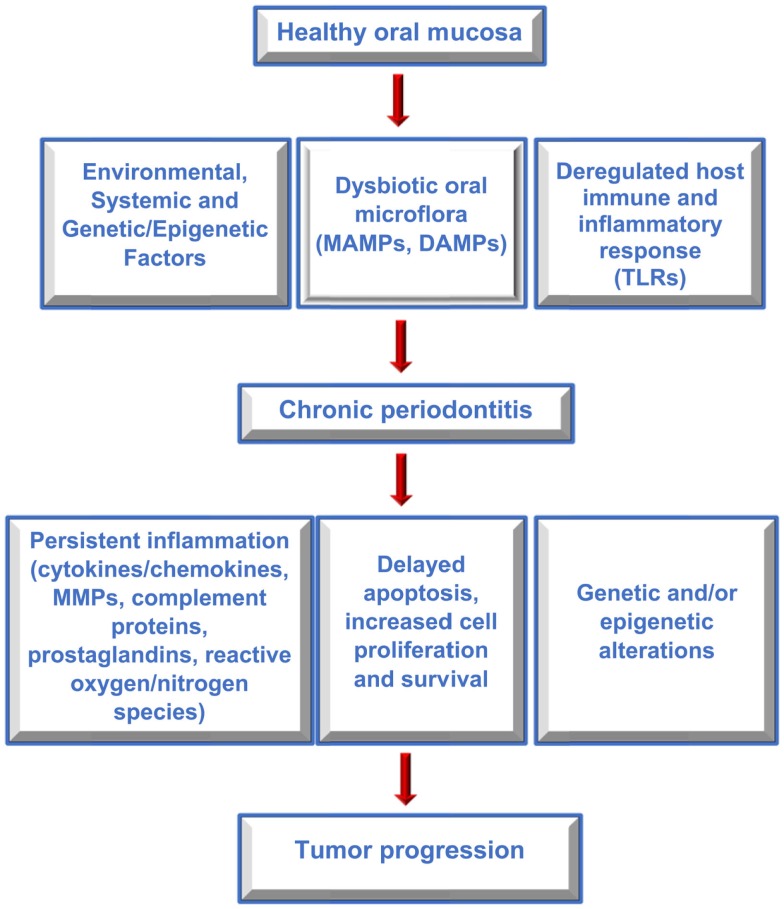
**Factors contributing to periodontitis and cancer**. Flow diagram outlining the interplay between host and environmental factors that modify the oral microenvironment, contribute to periodontitis and oral cancer, and which may link the two diseases.

**Figure 3:**
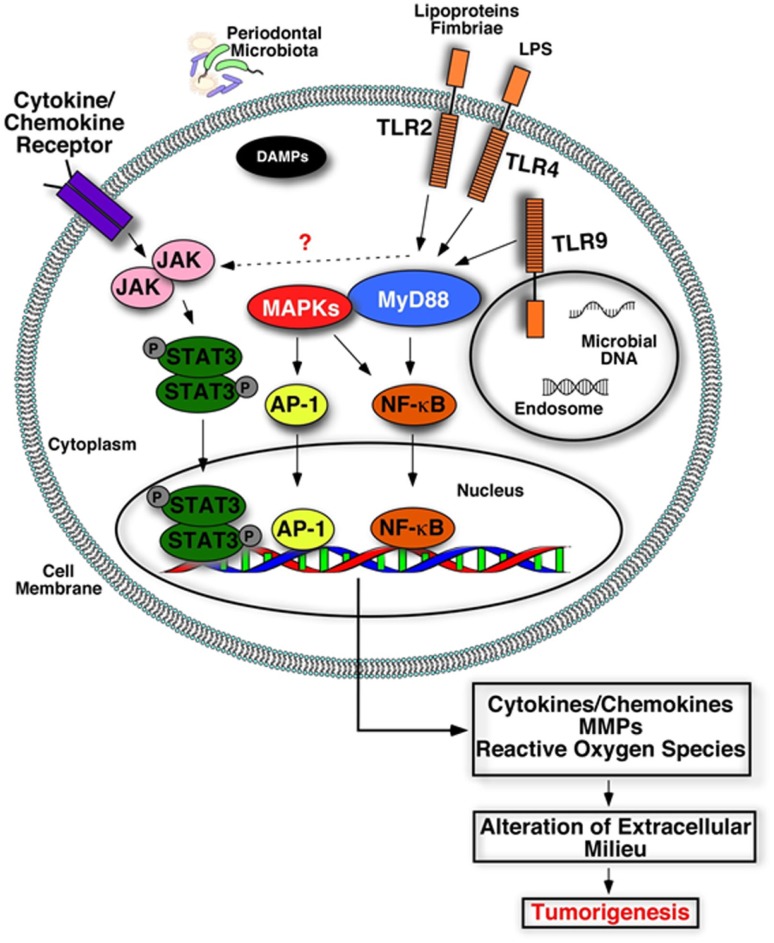
**Plausible biological mechanisms that may link deregulated inflammation and cancer**. Engagement of microbial components (LPS, lipoproteins, nucleic acids) or damage-associated molecular patterns (DAMPs) with their receptors (TLRs) triggers activation of inflammatory signaling cascades and increases production of inflammatory mediators, tissue destructive enzymes (MMPs), and reactive oxygen/nitrogen species. Accumulation of these host-derived factors within the mucosa due to deregulated inflammation may alter and create a favorable oral microenvironment that promotes tumorigenesis.

Several studies have reported an association between chronic periodontitis and cancer both in the oral cavity as well as at distant sites (22, 79, 80). Studies that have investigated the association between periodontal disease and oral cavity cancer are summarized in Table 1. While plausible biological mechanisms exist that can link the two diseases (Figures 2 and 3), the exact etiology is yet to be established. Tezal and coworkers reported that patients with periodontitis had a 5.23-fold increased risk of tongue cancer for each millimeter of alveolar bone loss (81). A further case-control study suggested a similar trend, with a stronger association between periodontitis and oral cavity cancer, compared to oropharyngeal or laryngeal tumors (82), and this was still noticeable in non-smokers. In addition, SCC lesions were more likely to be poorly differentiated in periodontitis patients compared to patients without periodontitis. Furthermore, a study of base-of-tongue cancers indicated that patients with HPV-positive tumors had greater bone loss than those with HPV-negative lesions (83), with the authors concluding that chronic periodontitis may influence HPV infection. Interestingly, a cross-sectional study revealed a relationship between alcohol consumption (a recognized risk factor for oral cavity cancer development) and clinical attachment loss (84). Thus, it may be possible that alcohol promotes oral carcinogenesis through multiple mechanisms, including potentiation of periodontal inflammation. As outlined above, constant polymicrobial insult and production of inflammatory mediators drive the initiation, progression, and persistence of periodontitis lesions. Chronic exposure to microbial and host-derived products can likely modify the oral microenvironment and possibly distant tissues, promoting carcinogenesis or at least increasing susceptibility. Ongoing research efforts focus on the working hypotheses that the chronic periodontitis may be linked with oral cancer risk either through direct toxic effects of the oral microbiome and associated byproducts and/or through the indirect effect of chronic oral inflammation. The next section will give an overview of potential biological mechanisms related to persistent, chronic periodontal disease that may promote cancer development in the oral cavity.

**Table 1:** **Studies investigating the link between oral cancer and periodontal disease**.

Author	Study design	Oral health criteria	Study population	Results
Bundgaard et al. (85)	Case-control	Missing teeth	161 patients and 400 controls (Denmark)	Significantly increased risk of developing oral SCCA for patients with fewer than 15 teeth
Rezende et al. (86)	Case-control	CPITN^a^ and DMFT^b^	50 patients and 50 controls (Cuba)	76% of subjects in cancer group showed greater than 6 mm pockets compared to 10% of control group
Garrote et al. (87)	Case-control	Missing teeth	200 patients and controls (Cuba)	Significantly increased risk of oral cancer for patients missing 16 or more teeth
Hiraki et al. (88)	Case-control	Missing teeth	429 patients and 10,480 controls (Japan)	Significantly increased risk of head and neck cancer with decreased number remaining teeth
Marshall et al. (89)	Case-control	Missing teeth	290 patients and controls (United States)	Significantly increased risk of oral cancer with loss of 11 or more teeth
Michaud et al. (90)	Cohort	Self-reported history of periodontal disease (confirmed by radiographs and missing teeth)	118 patients (United States)	No significant increase in risk for oropharyngeal cancer with history of periodontal disease or increased number of tooth loss
Rosenquist et al. (91)	Case-control	Missing teeth	132 patients and 320 controls (Sweden)	Significantly increased risk of oral and oropharyngeal cancer for missing over 20 teeth
Tezal et al. (92)	Cohort	Clinical attachment loss (CAL)	131 oral tumors and 323 oral pre-cancerous lesions (United States)	Significantly increased risk of oral tumor and pre-cancerous lesion with >1.5 mm clinical attachment loss
Tezal et al. (81)	Case-control	Alveolar bone loss	51 cases and 54 controls (United States)	Significantly increased risk of tongue cancer with increased alveolar bone loss
Tezal et al. (82)	Case-control	Alveolar bone loss	266 patients and 207 controls (United States)	Significantly increased risk of oral cavity SCC with periodontitis
Tezal et al. (93)	Case-control	Alveolar bone loss	124 head and neck SCC patients (United States)	Periodontitis is associated with tumor HPV status
Wen et al. (94)	Cohort	Medical records from insurance claims	96,375 gingivitis and 51,791 periodontitis cases (Taiwan)	Significantly increased risk of oral cancer with the history of periodontitis
Zheng et al. (95)	Case-control	Missing teeth	404 subjects and controls (United States)	Significantly increased risk of oral cancer with increased missing teeth

*^a^WHO guidelines of community periodontal index of treatment needs (depth of periodontal pockets on scale of 1–4)*.

*^b^WHO guidelines of decayed, missing, and filled teeth*.

## The Contribution of the Oral Microbiome

As mentioned above, a number of factors are considered to play an important role in the genesis of oral cancer (96), including tobacco and alcohol use, dietary factors such as saturated fat and fruit/vegetable intake, and microbiological agents. While tobacco and alcohol are recognized as being primary etiologic factors for oral squamous carcinogenesis, there is a worrying trend in the incidence of oral cancer in patients who have never used these agents (97), raising the possibility that other factors may have a major role in tumor progression in these subjects. Furthermore, many users of tobacco and alcohol do not develop malignant oral disease, suggesting that additional events and/or cofactors are of importance.

A diverse group of microbial species inhabit the oral cavity, including bacteria, viruses, and fungi. For many years, it has been suggested that oncogenic human papillomaviruses may contribute to oral squamous carcinogenesis (98–101), similar to their role in cervical cancer. However, more recent studies have indicated that HPV may be more closely related to particular subsets of disease, specifically oropharyngeal and tonsillar carcinomas (102–105).

In addition to a possible viral etiology of a subset of oral cancers, bacteria have also been implicated in tumor development. For many years, *Treponema pallidum* has been associated with oral squamous carcinogenesis (106, 107). While the incidence of syphilis has declined markedly over the last century, it may still be involved in a small number of cases (108). However, poor oral hygiene has also been documented as a risk factor for development of oral malignancy (85, 89, 91, 109, 110), raising the possibility that some of the many other bacterial species present in the oral cavity may be of importance. For example, in a study of bacterial species associated with oral SCC, Nagy and coworkers reported a significantly higher number of anaerobic periodontal bacteria associated with malignant lesions compared to normal mucosa, including periodontopathogenic *Prevotella* and *Porphyromonas* species (28). Another study investigated the relationship between salivary micro-organisms and oral cancer (27). Interestingly, these authors reported increased levels of bacterial species associated with SCC, including *Prevotella*, *Porphyromonas*, and other species, and demonstrated that the levels of at least three salivary bacterial species were predictive of around 80% of SCCs. Whether this relationship is merely associative or indicates a role for oral bacteria in neoplastic progression remains to be determined.

The idea of a bacterial etiology for human cancer is by no means limited to the oral cavity. Indeed, it is readily apparent that *H. pylori* infection is causally associated with malignancies of gastric epithelia (111, 112), leading to the categorization of this organism as a WHO class I carcinogen. *H. pylori* causes inflammation of the gastric mucosa, likely by inducing gastric epithelial cells to secrete IL-8, which results in recruitment of inflammatory cells to the site of infection (113). Moreover, IL-8 has the capacity to stimulate proliferation of epithelial cells, at least in part through transactivation of the EGF receptor (114). Further, as mentioned above, IL-8 and other chemokines and their receptors have been implicated in tumor development and metastatic progression in a number of human malignancies (115). This may well be true for oral cavity cancers and that oral bacterial species have the capacity to induce parallel events. In fact, a recent investigation reported that increased IL-8 expression in OSCC significantly correlated with increased serum IL-8 levels and poor clinical outcomes. The study also showed that IL-8 enhanced generation of CD163-positive M2 macrophages, and that CD163 positivity at the tumor invasion front correlated with significantly worse overall survival and disease-free survival in OSCC (116). In further support of the possible contribution of the oral microbiome to tumorigenesis, it was reported that hepatocyte growth factor (HGF) can be induced in fibroblasts by oral bacteria as well as by cytokines (117), while production of CCL20 was upregulated by *Actinobacillus actinomycetemcomitans*, *E. coli* LPS, and TNF-α (118). This provides a link between the oral microflora and factors which are known to influence both the proliferation and migration of epithelial cells. Additionally, studies by Sakamoto and coworkers investigated bacterial colonization of oral tumors and cervical lymph nodes (119, 120). Viable bacteria were recovered from both tumor and lymph nodes, with involved nodes showing higher colonization than uninvolved nodes (120). It was suggested that disruption of the integrity of the epithelial layer by the primary tumor might facilitate bacterial entry and subsequent lymphatic drainage. If, indeed, oral bacteria can colonize and survive in lymph nodes, they might stimulate the production of factors that could aid survival and proliferation of any tumor cells in the immediate environment. Furthermore, the association of *F. nucleatum*, another periodontal bacterial species, with colorectal cancer also supports the notion that oral pathogens can obtain access to distant sites and may participate in tumorigenesis in other organs as well (17, 121). *F. nucleatum* is one of the most frequently identified bacteria both in healthy gingiva as well as periodontitis sites and can also induce production of cytokines and chemokines (121).

Another alternative may be that oral bacteria invade tumor cells at the primary site, influencing their biological behavior. *P. gingivalis* has the ability to invade and propagate intracellularly (122), including in gingival epithelial cells (123). It can also delay apoptosis in gingival epithelial cells through various different mechanisms, such as by activating the JAK1/STAT3/Akt pathway, downregulating caspase-3 and caspase 9 expression, upregulating microRNAs (specifically miR-203), leading to suppression of SOCS3, preventing ATP-dependent apoptosis (124–128). Programed cell death is one mechanism through which cells avoid replicating if DNA damage is excessive and cannot be repaired. Thus, by interfering with this key regulatory process *P. gingivalis* may promote carcinogenesis. Furthermore, *P. gingivalis* also possesses both anti- and pro-inflammatory activities thereby contributing to dysbiotic microflora (129). This feature also gives the bacterium a unique ability to manipulate the immune system for its own survival. *P. gingivalis* can also promote invasion of oral cancer cells by activating the ERK1/2-Ets1, p38/HSP27, and PAR2/NF-κB pathways to induce MMP9 (130). Increased IL-6 and IL-8 production was also reported in HSC-3 and H413 oral cancer cells in response to *P. gingivalis* challenge (131). Another important way in which *P. gingivalis* might contribute to tumor progression is through its ability to suppress angiostatic chemokines. In a key study (132), Jauregui et al. found that this bacterium could suppress the secretion of CXCL9, CXCL10, and CXCL11. Thus, the removal of their regulatory activity could promote neovascularization and enhance tumor growth.

While the bacterial etiology of periodontitis is well established, the contribution of viruses to the pathogenesis of periodontal disease has also been supported by a number of studies (133–137). It is likely that viral/bacterial interactions in the oral cavity can further modulate the oral microenvironment. For example, it has been shown that Herpes simplex virus type 1 (HSV-1), Epstein-Barr virus (EBV), and human cytomegalovirus-infected periodontitis lesions harbor elevated levels of periodontal pathogens. It was also reported that EBV can reside in gingival epithelial cells, which may serve as an oral reservoir for virus infected cells. Importantly, epithelial EBV infection was significantly increased in chronic periodontitis (138). Additionally, both HPV and EBV have been implicated in head and neck carcinogenesis. As mentioned above, a study among patients with base-of-tongue cancer suggested a synergy between periodontitis and HPV status (82), and chronic periodontitis was also associated with HPV positive tumor status in patients with incident primary squamous cell carcinoma of the oral cavity, larynx, and oropharynx (93). The biological and molecular mechanisms defining how bacterial/viral interactions modify the tumor microenvironment and lead to disease initiation or progression are poorly understood, and are an area of active investigation. A plausible possibility is the activation of innate immune sensors such as TLRs (see below). For example, TLR-2, TLR-4, and TLR-9 can be stimulated by oral bacterial species, whereas viruses activate TLR-3, -7, -8, and -9. Thus, there would seem to be scope for synergistic activation of multiple pathways in response to different components of the oral microbiome, resulting in gene activation and production of secreted mediators into the extracellular millieu. However, this is likely to be a complicated scenario as some studies have reported that activation of specific TLRs (for example, TLR-3 and -7) may trigger apoptosis rather than pro-survival and pro-oncogenic mechanisms (139–141).

## The Contribution of Chronic Inflammation

Disease progression in periodontitis is a complex process that involves the interaction of multiple components of the host immune response and the oral microbiome, as outlined above. The engagement of MAMPs and DAMPs with their cognate receptors releases inflammatory mediators that aid in the development of an efficient innate immune response to eliminate the pathogen and coordinate development of an adaptive immune response. However, the impaired tissue homeostasis and deregulated inflammation in periodontitis exposes the local and systemic tissues to noxious metabolic products including living and necrotic cells, cytokines, chemokines, prostaglandins, MMPs, reactive oxygen and nitrogen radicals. Accumulation of these host-derived factors may likely modulate the tumor microenvironment by causing DNA damage, promoting epigenetic and genetic alterations, increasing angiogenesis, cell survival, proliferation, migration, and inhibiting apoptosis (Figures 2 and 3). Altered cells may eventually promote production of more inflammatory mediators, further enhancing inflammation and contributing to cancer pathogenesis.

One mechanism through which host cells detect microbial challenge is through engagement of microbial components with innate receptors, specifically TLRs. These receptors can interact with components of bacteria, viruses, and fungi (142). Several cell types found in periodontal tissues express TLRs, and each receptor is involved in the sensing of distinct microbial products. TLRs are type I trans-membrane proteins composed of an extracellular leucine-rich repeat (LRR) domain involved in ligand recognition, a trans-membrane domain, and a toll-interleukin 1 receptor (TIR) domain involved in signaling. The signaling pathways activated by TLRs engage adaptor molecules that are recruited by TIR/TIR domain interactions. These include the myeloid differentiation primary response gene (MyD88), the TIR domain-containing adaptor protein (TIRAP, also known as MAL), TIR domain-containing adaptor inducing interferon-β (TRIF), and the TRIF-related adaptor molecule (TRAM). MyD88 is essential for signaling through all TLRs, except TLR3, and is involved in early activation of NF-κB and MAPKs, leading to pro-inflammatory gene expression. As well as immune cells and oral keratinocytes, oral cancer cells also express TLRs (143–146). Thus, tumor cells or their premalignant counterparts may respond to direct stimulation by oral microbes or their byproducts, activating pro-inflammatory, pro-proliferative, and pro-migratory signaling pathways.

Of high relevance to carcinogenesis, an interesting study by Lappin and colleagues (147) determined plasma concentrations of CXCL5 and IL-6 in systemically healthy subjects with or without periodontitis, and who either did or did not smoke. They found significantly higher levels of circulating CXCL5 in smokers with periodontitis, which correlated with probing depth, attachment loss, and tobacco consumption. Given the potential function of CXCL5 as a pro-angiogenic factor (148), together with its role to promote tumor cell growth and motility (60), high circulating levels of CXCL5 could potentially act to promote tumorigenic progression in concert with local factors.

The loss of periodontal tissue integrity due to chronic inflammation aids viral and bacterial survival and persistence, as well as enhancing the inflammatory response. It is therefore plausible to hypothesize that prolonged inflammation in periodontal tissues due to activation of innate sensors by periodontal microbial products or DAMPs may promote a tumor-favorable environment by exposing the tissues to multiple cytokines/chemokines. In fact, emerging evidence also supports this concept (149). Increased expression of host innate receptors has been reported in periodontitis lesions compared to healthy sites (150–153). Among these, TLR9 has been implicated in oral squamous cell carcinogenesis (149). TLR9 is an intracellular sensor that can activate a range of cell types such as macrophages, dendritic cells, PMNs, B cells, and epithelial cells through recognition of bacterial and viral DNA sequences that are released following phagocytosis. TLR9 activation can trigger production of multiple inflammatory mediators in response to periodontal pathogens, including IL-8 production from gingival epithelial cells and macrophages (154–157). TLR9 expression was also reported to be significantly elevated in the tissues of oral squamous cell carcinoma as well as periodontitis, and increased receptor expression was correlated with increased tumor size and clinical stage (153, 158, 159). *In vitro* studies revealed that activation of TLR9 can mediate oral cancer cell migration by up-regulating MMP2, and tumor cell proliferation by up-regulating cyclin D1 expression, both in an AP-1-dependent manner (159, 160). Increased IL-1α and IL-6 production in OSCC cells treated with a TLR9 agonist was also reported (160). It is therefore possible that TLR9 activation can contribute to tumorigenesis by enhancing inflammation and through modulating cell cycle progression. Another clinical investigation reported a strong correlation between TLR2, -4, and -9 expression and increased tumor invasion in oral tongue squamous cell carcinoma (161). This study also revealed that increased TLR9 expression correlated with advanced tumor size, while high TLR5 expression was associated with a lower tumor grade. It is important to emphasize that all of these receptors are also activated by periodontal bacteria in the course of periodontal disease and trigger production of cytokines and chemokines (162) through NF-κB, AP-1, and MAPKs. Among these molecules, NF-κB expression and activity is often elevated in oral cancers, with protein levels gradually increasing as the lesion progresses from premalignant to invasive form (163–165). Further, aberrant function of NF-κB has also been reported to stimulate STAT3 activation by an autocrine/paracrine mechanism in SCC, suggesting crosstalk between these signaling pathways (166). STAT3 is a critical mediator of the pro-angiogenic and immunosuppressive activities of myeloid cells in the tumor microenvironment, and it can lead to enhanced cell cycle progression and neovascularization, thus promoting tumor growth (167, 168). Collectively, these findings support the hypothesis that aberrant activity of multiple innate receptors and inter-related downstream signaling pathways contribute to oral tumorigenesis. Intriguingly, STAT3 and NF-κB lie downstream of TLR-mediated inflammatory cascades including those activated by TLR-2, -4, and -9 (169). It remains to be determined whether STAT3 signaling is triggered as a result of direct stimulation of these receptors or as a result of the production of cytokines, such as IL-6, and development of a positive feedback loop.

In summary, it is likely that deregulated receptor activity within the oral cavity due to chronic periodontitis may be associated with tumor progression, possibly as a result of exposure of the mucosa to cytokines, chemokines, MMPs, and reactive oxygen (or nitrogen) radicals. Accumulation of these host-derived factors are likely to modulate the tumor microenvironment by causing DNA damage, promoting epigenetic and genetic alterations, increasing angiogenesis, cell survival, proliferation, migration, and inhibiting apoptosis. Altered cells may eventually promote production of more inflammatory mediators, further enhancing inflammation thereby contributing to cancer pathogenesis (Figure 3). Still, the role of TLRs in cancer is ambiguous, as they can either mediate signaling leading to inhibition of apoptosis and altered cell proliferation, or trigger immune responses to cancer. While the observations outlined above provide some evidence for plausible mechanisms linking cancer and periodontal inflammation, future studies are warranted to delineate the specific cellular and molecular pathways that may precipitate tumorigenesis in the oral cavity.

## Concluding Remarks

**Figure 4:**
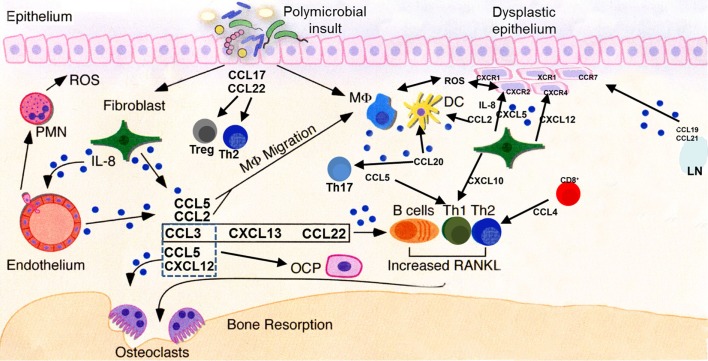
**Key chemokine functionalities in periodontitis and oral carcinogenesis**. A dysbiotic oral microflora triggers inflammatory processes in the oral epithelium. Release of chemokines, among other molecules, results in progression (or suppression, in some cases) of the inflammatory process and stimulation of both innate and adaptive immune responses through recruitment of cellular mediators. Persistent inflammation extends deeper into the tissues, subsequently leading to osteoclast activation and subsequent destruction of alveolar bone. Multiple chemokines involved in the periodontal inflammatory process may stimulate their cognate receptors present on normal, dysplastic, or malignant epithelial cells, deregulating cellular growth, and promoting the motile phenotype. Pro-angiogenic chemokines, such as IL-8 and CXCL5, act upon endothelial cells to promote neovascularization of developing tumors. LN, lymph node; ROS, reactive oxygen species; PMN, neutrophil polymorph; OCP, osteoclast precursor; DC, dendritic cell; MΦ, macrophage.

Emerging evidence argues that mutual interactions between host cells and the oral microbiome (bacteria and/or viruses) in the course of chronic periodontal inflammation likely creates a tumor-favorable microenvironment that may promote cancer development and progression (Figure 4). However, studies of the biological and biochemical mechanisms to explain the observed associations between oral cancer and periodontitis are at an early stage, and future investigations are warranted to determine the etiology and to delineate the molecular pathways involved. While the studies are underway exploring the possible cause–effect relationship between periodontal inflammation and oral cancer, it is imperative for health care professionals to make their patients aware of the current evidence that there might be a link between periodontal disease and cancer development. Thus, maintaining good oral health should be considered as part of a healthy lifestyle, not only to prevent tooth loss but also for overall systemic health. Individuals who exhibit periodontal disease and who also have other risk factors related to their lifestyle or family history may benefit from more frequent periodontal maintenance visits to help maintain the infection and inflammation at a minimal level. In general, patients should be encouraged to adopt positive lifestyle habits such as regular physical and dental visits, meticulous oral hygiene, cessation of smoking, healthy eating habits, regular exercise, and elimination of other risks that may predispose to malignant transformation. It is also imperative for dental and medical professionals to communicate with each other and work as a team to manage their patients and reduce or eliminate possible risks, resulting in better overall oral and systemic health.

## Conflict of Interest Statement

The authors declare that the research was conducted in the absence of any commercial or financial relationships that could be construed as a potential conflict of interest.
